# Role of* Porphyromonas gingivalis* HmuY in Immunopathogenesis of Chronic Periodontitis

**DOI:** 10.1155/2016/7465852

**Published:** 2016-06-15

**Authors:** P. C. Carvalho-Filho, I. S. Gomes-Filho, R. Meyer, T. Olczak, M. T. Xavier, S. C. Trindade

**Affiliations:** ^1^Odontology Course, Bahiana School of Medicine and Public Health, 41150-100 Salvador, BA, Brazil; ^2^Department of Periodontics, Feira de Santana State University, 44036-900 Feira de Santana, BA, Brazil; ^3^Department of Biointeraction, Federal University of Bahia, 40110-100 Salvador, BA, Brazil; ^4^Faculty of Biotechnology, University of Wroclaw, 50-383 Wroclaw, Poland

## Abstract

Periodontitis is a multifactorial disease, with participation of bacterial, environmental, and host factors. It results from synergistic and dysbiotic multispecies microorganisms, critical “keystone pathogens,” affecting the whole bacterial community. The purpose of this study was to review the role of* Porphyromonas gingivalis* in the immunopathogenesis of chronic periodontitis, with special attention paid to HmuY. The host response during periodontitis involves the innate and adaptive immune system, leading to chronic inflammation and progressive destruction of tooth-supporting tissues. In this proinflammatory process, the ability of* P. gingivalis* to evade the host immune response and access nutrients in the microenvironment is directly related to its survival, proliferation, and infection. Furthermore, heme is an essential nutrient for development of these bacteria, and HmuY is responsible for its capture from host heme-binding proteins. The inflammatory potential of* P. gingivalis* HmuY has been shown, including induction of high levels of proinflammatory cytokines and CCL2, decreased levels of IL-8, and increased levels of anti-HmuY IgG and IgG1 antibodies in individuals with chronic periodontitis. Therefore, the HmuY protein might be a promising target for therapeutic strategies and for development of diagnostic methods in chronic periodontitis, especially in the case of patients with chronic periodontitis not responding to treatment, monitoring, and maintenance therapy.

## 1. Introduction

Periodontal diseases are among the most common chronic inflammatory diseases in humans [1]. They comprise a number of inflammatory and infectious conditions caused by the inflammatory host response to bacteria in the supragingival and subgingival biofilm. The presence of periodontal pathogens may lead to an imbalance in the periodontal environment, and the subsequent host innate and adaptive immune response may lead to soft and/or hard tissue destruction. Periodontal pathogens composing a biofilm can injure periodontal tissues by way of the inflammatory response. Periodontitis may affect the gingiva, causing gingivitis, or may progress to the supporting periodontium, potentially affecting tooth mobility, which may lead to tooth loss [2]. Localized and aggressive forms of periodontitis are associated with* Aggregatibacter actinomycetemcomitans*, while chronic forms of generalized disease involve other bacteria, including* Porphyromonas gingivalis*,* Tannerella forsythia*,* Prevotella intermedia*, and* Treponema denticola* [3]. Periodontal diseases are modulated by the immune response and can be a risk factor for systemic disorders. Current evidence supports the importance of several factors increasing onset and progression of periodontal diseases, including smoking [4]. Tobacco use can also lead to diabetes mellitus, which may influence inflammatory changes in periodontal tissues. Other potential interactions with periodontal disease are still being investigated, such as those involving obesity, hormonal changes, cardiovascular and respiratory diseases, and adverse pregnancy outcomes [5, 6].

Several recent studies have proposed a new model of pathogenesis for periodontitis, pointing to a synergistic and dysbiotic microbial community responsible for the initiation of periodontal diseases, instead of the action of selected periodontal pathogens [7–9]. Bacteria termed “keystone pathogens,” found in low abundance under healthy conditions, can destabilize the community and cause the development of dysbiosis. The best-documented example of such pathogens is* P. gingivalis*, an anaerobic Gram-negative coccobacillus which belongs to the Bacteroidaceae family. In the natural environment,* P. gingivalis* is a constituent of the multispecies biofilm [10, 11]. The bacterium can also enter gingival epithelial and immune cells, remain viable and capable of spreading among cells [12–14], and spread systemically to other tissues [15–19]. A number of studies have demonstrated that* P. gingivalis* is localized in various subcellular compartments of host cells, including cytoplasm, endosomes, and autophagosomes. It has been found that the bacterium instead of trafficking to the endosomal pathway traffics to the autophagosome-like vacuoles and resides in vacuoles that resemble early and late autophagosomes, which may allow survival by blocking fusion with lysosomes [12, 20, 21]. Bacterial trafficking to the autophagic pathway allows protection from the host's defense mechanisms and acquisition of nutrients, which is especially beneficial for asaccharolytic* P. gingivalis*. Moreover, outer-membrane vesicles produced by* P. gingivalis* enter human cells via a lipid raft-dependent endocytic pathway, are routed to endosomes, and are sorted to lysosomal compartments [22, 23]. All these data suggest that this pathogen has the ability to invade host cells [24], which can be an escape mechanism from host defenses, favoring the microorganism's penetration in the bloodstream and thus acting systemically in the host body [25].

Important features of* P. gingivalis*-mediated chronic periodontitis include the ability of the bacterium to adhere to and invade host cells, disseminate through host cells and tissues, and subvert host immunological surveillance and defense mechanisms. However, virulence determinants of periodontopathogens that allow for efficient infectivity and promote synergy in the increase of virulence are still not clear. The aim of this review is to present the role that* P. gingivalis* antigenic determinants play in the immunopathogenesis of chronic periodontitis, with special attention paid to the* P. gingivalis* HmuY protein.

## 2. Immunopathogenesis of Chronic Periodontitis

While bacterial infection is the primary etiologic factor, it is not sufficient to induce the onset and progression of periodontitis. A localized inflammatory reaction is stimulated by bacteria components, resulting in activation of the host innate immune system. The innate response involves the recognition of microbial components by Toll-like receptors (TLRs) expressed by host cells in the infected microenvironment [26]. Activation of these cells leads to the release of proinflammatory cytokines and the recruitment of phagocytes and lymphocytes. The activation of T lymphocytes initiates an adaptive immune response, Th1, Th2, Treg, or Th17, whereas B lymphocytes also participate in this process via the production of antibodies [27].

CD4^+^ and CD8^+^ T cells become activated following the recognition of microbial components, and many functionally distinct subsets of these lymphocytes have been described, each expressing different cytokines and transcription factors. NF-kappaB (NF*κ*B) is a key transcription factor complex that appears to play a critical role in the regulation of an acute inflammatory response by activating a cascade of cytokines and producing other proinflammatory mediators, including adhesion molecules (e.g., ICAM-1, VCAM-1, and E-selectin), enzymes (e.g., COX-2, 5-LO, CPLA, and iNOS), cytokines (e.g., IL-1, TNF, IL-6, GM, and G-CSF), and chemokines (e.g., IL-8, RANTES, MCP-1, eotaxin, and MIP-1*κ*) [28–30]. The activation of NF*κ*B may be inhibited by NFKBIL1 expression [31]. Genetic variations in NFKBIL1 are associated with susceptibility to inflammatory and/or autoimmune diseases [32].

Activated naive CD4^+^ T cells may differentiate into either Th1 lymphocytes expressing T-bet transcription factor, IL-2, IFN-*γ*, and TNF-*β*, or Th2 lymphocytes, which express GATA-3 and IL-4, IL-5, and IL-13, or Th17 or ROR*γ*t lymphocytes expressing IL-17A, IL-17F, IL-21, and IL-22. The specific cytokines produced play a role in determining the inflammatory process. Recent analyses of cytokine profiles and transcription factors have shown that Th17, Tf-h, Th9, and Th22 profiles may become activated in periodontal diseases [33–38]. Effector T cells may become naive, recently activated, or become memory T cells that can be distinguished by cell surface markers [39].

Recent studies have demonstrated the involvement of IL-33 in the pathogenesis of periodontitis [40–42]. This cytokine has been described as an IL-1 family member that is expressed by many cell types following proinflammatory stimulation and participates in cell lysis mechanisms. Increased levels of IL-33 secreted in periodontal tissues can exacerbate the periodontal destruction induced by RANKL [40, 43]. Some studies have shown a positive association between elevated levels of IL-33 in periodontal tissue and periodontitis [44, 45]. However, this issue is still controversial and some authors have suggested that this cytokine plays a protective role by inducing a predominant Th2 profile [42]. Several studies have shown that also IL-18 can influence the pathogenesis of chronic periodontitis [46–51]. IL-18 is a potent proinflammatory cytokine with structural similarity to IL-1*β* [50]. In the presence of IL-12, IL-18 induces a Th1 response, whereas, in the absence of IL-12, a Th2 response is promoted [52]. CD4+ T cells also secrete proresorptive cytokines, such as IL-1, IL-6, and IL-17, and each of these cytokines stimulates the expression of the NF*κ*B ligand (RANKL) receptor activator in osteoblasts and fibroblasts, which promotes osteoclast formation via a contact-dependent process [53]. IL-10 may also be present in the microenvironment of periodontal lesions, promoting negative feedback by various cell types, including T cells, B cells, macrophages, NK cells, mast cells, and neutrophils. Additional negative effects of IL-10 include the modulation of IL-1, IL-8, IL-12, and TNF-*α* and the inhibition of phagocytosis [54].

Typically, the T cell repertoire contains CD4^+^ CD25^+^ T regulatory lymphocytes that control the autoreactive peripheral immune response [54]. The populations of CD4^+^ CD25^+^ T regulatory cells in periodontal disease have been shown to be higher in periodontitis compared to gingivitis [55]. T regulatory cells are responsible for mechanisms of tolerance, and the suppressive function of CD4^+^ CD25^+^ cells was found to be partly dependent on cell contact, suggesting that the human mucosa induces tolerance to different antigens [56]. T regulatory cells (Treg) [57, 58] and Th17 cells [59, 60] have been identified in periodontal tissues, suggesting the importance of immunoregulation in periodontal diseases. The clinical implications of these studies can be seen in the identification of Th1/Th2 and Treg/Th17 genes in peripheral blood and salivary transcriptomes. The recognition of their role in immunopathogenesis of periodontitis is being tested to evaluate their potential as markers of susceptibility for this disease [61].

## 3. Antigenic Determinants of* Porphyromonas gingivalis*

*P. gingivalis* strains 33277, 381, and A7436 can locally invade periodontal tissues and evade host defense mechanisms, using a range of virulence factors that disrupt the innate immune and inflammatory responses [62]. A variety of virulence factors of this bacterium, such as capsule components, lipopolysaccharides (LPS), fimbriae, proteases, and outer-membrane proteins, can promote immunogenicity by stimulating a mechanism of innate and adaptive immunity, in both the host humoral immune response and the host cellular immune response.

### 3.1. Gingipains

Among a variety of secreted and structural components that contribute to* P. gingivalis* virulence are arginine-specific gingipains (HRgpA and RgpB) and lysine-specific gingipain (Kgp) [8, 9, 12, 63]. Many studies have demonstrated that the protease activity of gingipains is responsible for a variety of virulent features of* P. gingivalis* and survival of this pathogen in host cells. However, contradictory roles of gingipains in the manipulation of host defense systems by* P. gingivalis* have been reported, since they act by both stimulating and inhibiting innate immune responses [64]. Moreover,* P. gingivalis* (strain HG66) HRgpA and Kgp, but not RgpB, mediate in a proteolytic-independent manner enhancement of production of proinflammatory cytokines in macrophages [65]. Such an effect may be caused by hemagglutinin/adhesion domains of Kgp and HRgpA. Gingipains produced by* P. gingivalis* participate in several mechanisms of host protein activation and deactivation by stimulating the expression of matrix metalloproteinases (MMPs) in fibroblasts [66–70]. MMPs are a group of zinc-dependent enzymes responsible for the degradation of the extracellular matrix during tissue renewal and also during inflammatory processes. They normally exhibit low levels of expression and activity in adult tissues but may be significantly increased in a variety of pathological conditions, thereby leading to tissue destruction by way of inflammatory disorders, tumor growth, and metastasis. Gingipains can cleave and degrade collagen and the connective tissue matrix under normal physiological pH and temperature conditions. When secreted, they can destroy periodontal tissues, degrade cytokines, deactivate the host's complement system components, and cleave various receptors, including CD14 on macrophages and CD4 and CD8 on T cells, thereby inhibiting host defense systems and facilitating* P. gingivalis* colonization [67].

### 3.2. LPS

The LPS of* P. gingivalis* is structurally different from the LPS of other Gram-negative bacteria and also has different immunogenic properties. It is recognized in innate host cells by TLR-2 [71–73] and can interact with TLR-2 and TLR-6 [73]. This unusual recognition pattern depends on the structural heterogeneity of lipid A [74], which enables connection to both TLR-2 and TLR-4 in association with CD14. Furthermore, a lipoprotein associated with LPS of* P. gingivalis* 381 strain (encoded in W83 under the PG1828 locus) appears to be involved in signaling through TLR-2, and its removal can markedly reduce the recognition of* P. gingivalis* strains 33277, A7436, 381, and W50 [75].

### 3.3. Other Proteins

Recently, an important role in immune signaling pathways was ascribed not only to* P. gingivalis* gingipains and LPS, but also to other proteins produced by this bacterium, including serine phosphatase (SerB), peptidyl arginine deaminase, nucleoside diphosphate kinase, and fimbriae (FIMA and MFA1), FimA, HemB, HbR, Hgp44, and RagB [76–78]. It has been shown that FimA signals through TLR2 and TLR4, and HemB signals through TLR4 [25, 74, 79, 80].

### 3.4. HmuY and Heme Uptake

The capacity of* P. gingivalis* to evade the host immune response and obtain nutrients in the microenvironment is directly related to its survival, proliferation, and infection, and one of the essential nutrients in the host environment is heme. To acquire heme as a main source of iron and protoporphyrin IX (PPIX),* P. gingivalis* strains A7436 and W83 have evolved sophisticated mechanisms that enable uptake of this compound bound to host hemoproteins [81–83]. To acquire heme,* P. gingivalis* uses hemagglutinins, proteases (particularly gingipains), lipoproteins, and outer-membrane receptors [67, 84–86]. It has been shown that Kgp and HRgpA can bind and subsequently cleave hemoglobin [87]. HmuR, an outer-membrane TonB-dependent receptor, is involved in heme transport through the* P. gingivalis* A7436, W83, and 381 outer membrane [88, 89], whereas HmuY is a membrane-associated heme-binding protein [90–92]. These proteins serve as the first example of a novel, two-component system, comprising an outer-membrane receptor and heme-binding hemophore-like protein.

The HmuY protein is associated with the outer membrane of the bacterial cell and outer-membrane vesicles through the lipid anchor [91, 92] and can be shed in an intact, soluble form through the limited proteolytic activity of* P. gingivalis* strains' (A7436, W83, and ATCC 33277) Kgp [86, 91]. HmuY may be functional in the form of dimers or tetramers. Both HmuY and HmuR appear to be essential for survival and growth of* P. gingivalis*, since* hmuY* and* hmuY-hmuR* mutant strains constructed in the A7436 strain showed defects in growth when heme and hemoglobin were used as the sole iron and PPIX sources [89]. Moreover, recent data demonstrated that HmuY is required for effective* in vivo P. gingivalis* growth and invasion of macrophages [93]. It has been shown that the HmuY protein is resistant to several proteases, including* P. gingivalis* gingipains and* P. intermedia* interpain A (InpA) [86, 94], as well as host proteases, including neutrophil elastase [95], which allows its distribution and persistence in the host environment.

### 3.5. Role of HmuY Protein in Cooperation/Competition with Other Periodontopathogens

Recent studies have shown syntrophy between different bacterial species within oral biofilm through mutual cooperation/competition for nutrient acquisition, especially between* P. gingivalis*,* T. denticola*,* P. intermedia*, and* T. forsythia*, which form a polymicrobial community and dominate the periodontal biofilm [96]. Recently, it has been shown that black pigmented anaerobes, including* P. gingivalis*, display a novel heme acquisition paradigm, whereby hemoglobin must be first oxidized to methemoglobin, facilitating heme release [97]. In the case of* P. gingivalis* strains W83 and W50, this involves the arginine-specific gingipain proteases [98] and in the case of* P. intermedia* the protease InpA [94, 99]. The bacteria are then able to fully proteolyze the more susceptible methemoglobin substrate to release free heme. Importantly, HmuY is resistant to proteolysis and thus able to cooperate with* P. gingivalis* gingipains or* P. intermedia* InpA to extract the heme from hemoglobin, previously converted proteolytically into methemoglobin.

### 3.6. HmuY and the Host Immune Response

Several reports have demonstrated that not only do* P. gingivalis* cells localize in various cellular compartments of host cells, including cytoplasm, endosomes, and autophagosomes [12, 20, 21, 100], but also outer-membrane vesicles produced by* P. gingivalis* enter human cells via a lipid raft-dependent endocytic pathway, are routed to endosomes, and then are sorted to lysosomal compartments [22, 23, 101]. After lysis of* P. gingivalis* cells or outer-membrane vesicles, their antigens can be identified and processed by antigen-presenting cells, such as macrophages, causing activation of the adaptive immune response and production of antibodies. We recently demonstrated that also HmuY protein, both the protein produced by live* P. gingivalis* cells and the soluble protein added to macrophage cultures either together with the* hmuY* mutant strain constructed in the A7436 strain or alone, is internalized in macrophages [93]. Therefore, the HmuY protein can be recognized by the host immune system during chronic periodontitis. Indeed, we found that anti-HmuY antibodies inhibited* P. gingivalis* growth and biofilm formation [91]. Antibodies directed against* P. gingivalis* A7436 and ATCC 33277 HmuY are highly specific for the purified HmuY protein and cell-bound HmuY and do not cross-react with HmuY homologs found in* P. intermedia* and* T. forsythia* [102]. This suggests that* P. gingivalis* ATCC 33277, W83, and A7436 HmuY can serve as a specific antigen for serum determination of antibodies raised against this bacterium in patients with chronic periodontitis [103].

The immunogenic potential of* P. gingivalis* HmuY has been further demonstrated through promotion of inflammation, mainly by inducing high levels of IL-1*β* and IL-6 [103, 104]. Furthermore, HmuY seems to participate in a delayed host response through the increase of IL-10 and IL-6 levels and IgG and IgG1 levels of anti-HmuY antibodies and decrease of IL-8 levels in individuals with chronic periodontitis [104, 105].* P. gingivalis* HmuY also evokes inflammatory responses in peripheral blood mononuclear cells (PBMC) derived from chronic periodontitis individuals, eliciting CCL2 and IL-18 as well as inhibiting NFKBIL-1 and IL-10 [105].

Other studies have shown that the protein formerly designated fibroblast-activating factor (FAF), previously characterized by Mihara and Holt [106] in* P. gingivalis* W50, W83, and ATCC 33277 strains, enhanced the proliferation of normal human gingival and skin fibroblasts, as well as umbilical vein endothelial cells. FAF induced higher levels of IL-6 production in human gingival fibroblasts compared to cells stimulated with* P. gingivalis* LPS and did not show action against human periodontal ligament cells [107, 108]. FAF has also been suggested to exert low phosphatase activity and participate in bone resorption. Interestingly, some amino acid sequences in peptides identified in FAF are identical to amino acid sequences in regions of the* P. gingivalis* HmuY protein [89].

The HmuY protein also seems to act in programmed cell death. PBMC stimulated with this protein appear to be unable to complete the process of apoptosis, resulting in death characterized by release of inflammatory cell content into the microenvironment, such as late apoptosis and necrosis, which can prolong the tissue destruction process [109]. Furthermore, the protein induces high levels of Bcl-2 in PBMC derived from individuals with chronic periodontitis, resulting in inhibition of apoptosis by enabling increased survival of T CD3^+^ cells. All these findings strongly suggest that HmuY is one of the important virulence factors that allow effective* P. gingivalis* infection of host cells [93].

## 4. Conclusions

**Figure 1:**
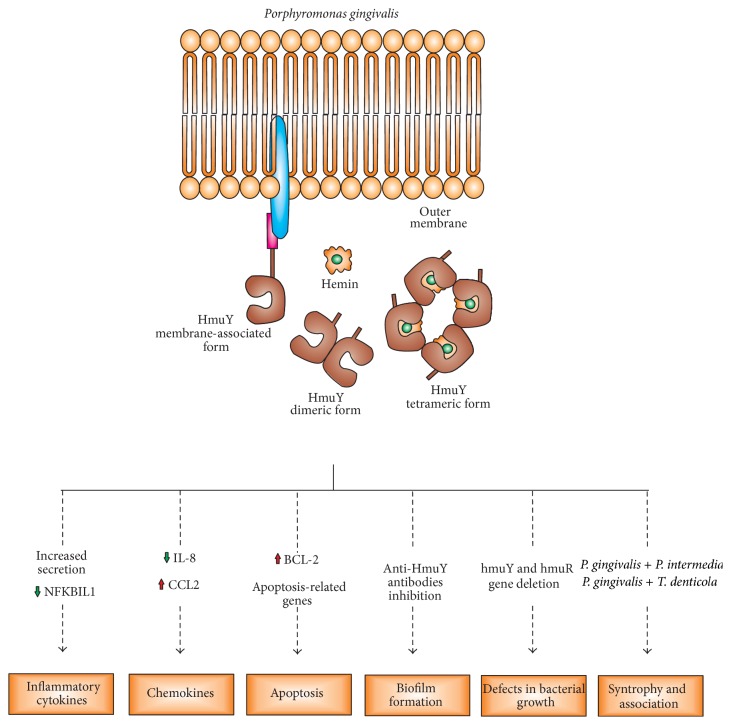
Schematic presentation of role of* P. gingivalis* HmuY in immunopathogenesis of chronic periodontitis.

*P. gingivalis*, a “keystone pathogen” of chronic periodontitis, is an important component of the oral microbiome and a highly adapted colonizer. The bacterium has the ability to evade host defense systems and interfere in relationships between other oral species that make up the microflora located in supragingival and subgingival periodontal biofilm, leading to chronic inflammation, a cell prosurvival profile, and consequent tissue damage observed in individuals with chronic periodontitis. Molecules produced by* P. gingivalis* play an important role in the immunopathogenesis of chronic periodontitis, acting in both innate and adaptive immunity. One may conclude that the HmuY protein is important, at least in part, for efficient* P. gingivalis* growth in the heme-limited host environment, where the HmuY hemophore sequesters heme from host hemoproteins, thus allowing for efficient infection of host cells. In conclusion,* P. gingivalis* HmuY might play an important role in the immunopathogenesis of chronic periodontitis to evoke inflammatory responses, inhibit apoptosis, and interact with other bacterial species in biofilm formation (Figure 1). Therefore, the HmuY protein could be a promising target for therapeutic strategies and for diagnosis of chronic periodontitis which does not respond to periodontal treatment, as well as in monitoring of maintenance therapy in individuals with chronic periodontitis.
